# Influence of Temperature on Seed Germination of Five Wild-Growing Tulipa Species of Greece Associated with Their Ecological Profiles: Implications for Conservation and Cultivation

**DOI:** 10.3390/plants12071574

**Published:** 2023-04-06

**Authors:** Stefanos Hatzilazarou, Elias Pipinis, Stefanos Kostas, Rafaela Stagiopoulou, Konstantina Gitsa, Eleftherios Dariotis, Manolis Avramakis, Ioulietta Samartza, Ioannis Plastiras, Eleni Kriemadi, Pepy Bareka, Christos Lykas, Georgios Tsoktouridis, Nikos Krigas

**Affiliations:** 1 Laboratory of Floriculture, School of Agriculture, Aristotle University of Thessaloniki, 54124 Thessaloniki, Greece; 2 Laboratory of Silviculture, School of Forestry and Natural Environment, Aristotle University of Thessaloniki, 54124 Thessaloniki, Greece; 3 Institute of Plant Breeding and Genetic Resources, Hellenic Agricultural Organization Demeter, P.O. Box 60458, Thermi, 57001 Thessaloniki, Greece; 4 Natural History Museum of Crete, University of Crete, 71409 Heraklion, Greece; 5 Thermokipia Athina, Metochi, 57500 Epanomi, Greece; 6 Laboratory of Systematic Botany, Department of Crop Science, Agricultural University of Athens, Iera Odos 75, 11855 Athens, Greece; 7 Department of Agriculture, Crop Production and Rural Environment, School of Agricultural Sciences, University of Thessaly, Volos, 38446 Magnesia, Greece; 8 Theofrastos Fertilizers, Irinis & Filias, Examilia Korithias, 20100 Korinthos, Greece

**Keywords:** tulips, seed dormancy, ecological profiles, biodiversity, bulbous plants, ecological requirements, threatened species

## Abstract

Although tulips are famous worldwide as ornamental plants, the knowledge about the seed germination of wild-growing species remains limited. The aim of the present study was to investigate the effect of temperature on seed germination of the local, wild-growing Greek endemics *Tulipa bakeri* and *T. goulimyi* and the sub-Balkan endemic *T. undulatifolia*, which are threatened with extinction, as well as the Mediterranean *T. australis* and the Asiatic *T. clusiana* naturalized on Chios Island (Greece). The germination responses at five constant temperatures (5, 10, 15, 20, and 25 °C) were assessed for all studied species in growth chambers under a 12:12 light–dark photoperiod. The ecological profile for each species was developed in R using open-source bioclimatic data; this was built to illustrate the abiotic environmental conditions of their wild habitats, to facilitate the examination of temperature effects on seed germination, and to facilitate their cultivation in artificial environments. The results indicated that the seed germination requirements of the studied species had a range-specific temperature dependence, reflecting their natural adaptation to local ecological conditions. Seed germination of *T. bakeri*, *T. australis*, and *T. clusiana* was observed only in a narrow range of very low temperatures (5–10 °C), whereas germination of *T. undulatifolia* and *T. goulimyi* occurred at temperatures between 5 and 15 °C. A temperature increase to 20 or 25 °C resulted in the absence of seed germination for all five Greek tulip species. The germinated seeds were planted in pots and bulblets were developed under greenhouse conditions. Seeds and bulblets constitute valuable genetic materials for the cultivation and ex situ conservation of these five Greek tulip species, three of which are threatened with extinction.

## 1. Introduction

Tulips are among the most popular ornamental plants and cut flowers produced worldwide. Botanical tulips refer to wild species that are used in the horticultural industry (garden plantings, cut flowers, or new breeding materials) due to their interesting characteristics (small size and unusual flower shapes and colors) and unique identities (local endemics from remote regions) associated with a natural rarity and limited availability (https://www.ahsgardening.org/gardening-resources/gardening-for-wildlife/ornamental-botanical-tulips, accessed on 22 February 2023). In recent years, wild tulip resources have attracted growing research attention, and currently, efforts are being made to domesticate, effectively reproduce, and cultivate in man-made settings several wild-growing tulips from China [1,2] and Greece [3]. Besides their interesting ornamental characteristics and unique identities, such wild phytogenetic resources exhibit a strong natural adaptability to cultivation settings and an enhanced resilience to pathogens, thus presenting as assets which can be exploited in the future for selective breeding strategies aimed at developing new tulip cultivars [2].

Although the asexual propagation of tulips is efficiently described in the literature (e.g., [4,5,6,7,8]), the knowledge about the requirements for the seed germination of numerous wild-growing tulip species remains limited. Low temperatures have been reported to promote the seed germination of several tulips such as *Tulipa systola* Stapf [9], *T. iliensis* Regel [10,11], and *T. kaufmanniana* Regel [12], as well as other wild-growing *Tulipa* spp. which are native to China [2]. This suggests the existence of seed dormancy, which in Chinese native tulips has been determined as a non-deep complex morphophysiological dormancy [2]. It has been suggested that the response of wild tulips to temperature may vary from species to species, especially in native range-restricted and rare varieties [2]. It is well known that the germination stage is crucial for any plant’s life cycle, and therefore, complex seed dormancy mechanisms may control and prevent the emergence of seedlings of wild species in a not favorable season or place [13,14,15]. In addition, seed germination may differ under controlled and natural conditions, with the latter showing comparatively lower germination percentages (in general, <50%) and seedlings that are easily affected by biotic and non-biotic interactions in their local environments [2]. Therefore, the study of the seed dormancy and germination behavior of wild-growing native tulips originated from different regions is essential to promote their ex situ propagation and conservation and to enable their sustainable exploitation.

The significant variation in temperature during different seasons in the Mediterranean regions is the most important environmental factor for seed germination as it regulates the appropriate timing for seedling establishment [16]. Furthermore, temperature is the environmental factor which is responsible for various changes in the dormancy of seeds [14]. Depending on their climatic origin, various species have different temperature requirements for seed germination [14]. The optimal germination temperature for Mediterranean species is usually relatively low, ranging from 5 to 15 °C, and germination in nature is often limited to early winter when rainfall is adequate [17,18,19,20]. This strategy ensures that plants may have a longer growing season period, lasting until the onset of typical Mediterranean summer droughts.

Considering the conservation needs due to the threatened and/or protected status of all the Greek tulips and the potential of Greek *Tulipa* spp. to be introduced as new cultivated crops [3], focus should be given to the study of the abiotic factors prevailing in their natural habitats. Itis particularly important to conceive a proxy of species-specific tolerance in terms of temperature and climatic conditions regarding future cultivation for ex situ conservation purposes or in the frame of sustainable exploitation strategies [21,22,23,24,25]. To this end, either GIS-derived [21,22] or R-derived species-specific ecological profiles [25,26] can be employed to generate the necessary information concerning the range of abiotic factors prevailing in the natural habitats of these bulbous species, thus facilitating their seed dormancy break and germination, pilot ex situ cultivation, and in situ reintroduction actions to enhance declining wild-growing populations [25,27].

In this framework, the present study aimed to investigate how temperature affects the germination of Greek tulip seeds under controlled conditions, thus determining, for the first time, germination protocols for 5 of the 15 members of the genus *Tulipa* that are wild-growing in Greece [21]. Species-wise, the study herein is focused on three threatened species, including the local Cretan endemic *T. bakeri* A. D. Hall; the local endemic of the Peloponnese, Kythira, Antikythera, and Crete islands, *T. goulimyi* Sealy & Turrill; and the sub-Balkan local endemic *T. undulatifolia* Boiss. [3,28]. In addition, the study also examines species of a wider distribution range, including the Mediterranean *T. australis* Link and the Asiatic *T. clusiana* Redouté, which is naturalized on Chios Island (Greece). The germination requirements for these tulip species have not been studied before, and therefore, this investigation sheds light on the sexual propagation of these species, assuming that they would require relatively low temperatures for their germination due to extant dormancy. To associate the results of the seed germination trials with environmental conditions prevailing in the wild habitats of the studied species, we investigated the ecological preferences of the five wild-growing Greek tulip species in terms of temperature and precipitation regimes based on all their known Greek localities to date. In this way, this study tried to unveil the seasonal bioclimatic preferences of each of the studied species with the aim to better understand their natural life cycle to facilitate their ex situ conservation and cultivation in artificial settings. To this end, we additionally documented the bulblet size of the derived seedlings after their first year of ex situ development.

## 2. Results

### 2.1. Ecological Profiling of Studied Species

**Figure 1:**
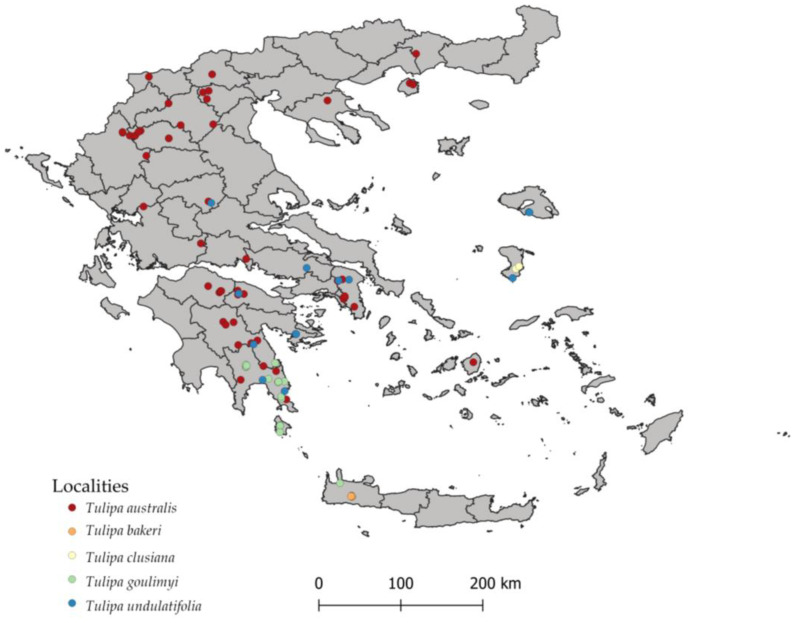
Overall known distribution of *Tulipa australis, T. bakeri, T. clusiana, T. goulimyi*, and *T. undulatifolia* in Greece. Red dots: distribution of *T. australis* (71 localities); orange dots: distribution of *T. bakeri* (11 localities); yellow dots: distribution of *T. clusiana* (2 localities); green dots: distribution of *T. goulimyi* (25 localities); blue dots: distribution of *T. undulatifolia* (24 localities).

Members of the genus *Tulipa* can be found across different areas both in mainland and insular Greece. Figure 1 presents the overall known distribution of *T. australis, T. bakeri, T. clusiana, T. goulimyi*, and *T. undulatifolia* in Greece to date.

**Figure 2:**
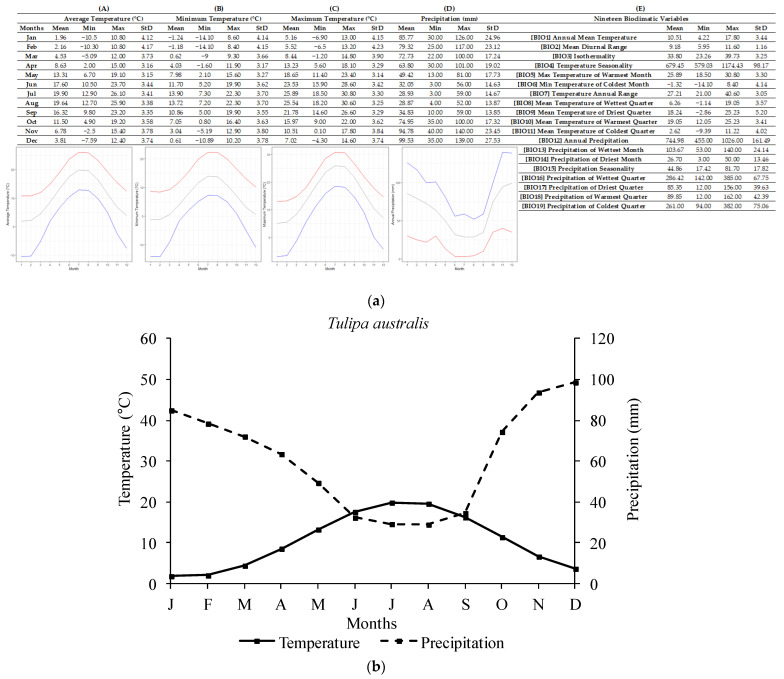
R-derived ecological profile (**a**) and Walter–Lieth climate diagram (**b**) of *Tulipa australis* based on 55 Greek localities known to date (*n* = 55). For (**A**–**E**), minimum, maximum, and average values and standard deviations are represented in different columns. Line graphs (**A**–**C**) illustrate the minimum (blue), the maximum (red), and the mean (grey) monthly temperature (°C) and the line graph (**D**) illustrates the minimum (red), maximum (blue), and mean (grey) monthly precipitation (mm). (**A**) shows the minimum temperature in each month (°C), (**B**) shows the maximum temperature in each month (°C), (**C**) shows the average temperatures per month (°C), (**D**) shows the precipitation in each month (mm), and (**E**) shows the values for each of the 19 bioclimatic variables.

The ecological profile of *T. australis* (Figure 2) has been compiled based on 55 localities of high accuracy which were scattered mainly across the Greek mainland. For *T. australis* sites, the annual average temperature recorded was 10.43 ± 3.37 °C, with the lowest annual average temperature being 4.22 °C and the highest being 17.80 °C. The annual precipitation recorded was 740.15 ± 163.08 mm and the lowest and highest annual values were 455 mm and 1026 mm, respectively. The lowest air temperatures were recorded in January (−1.37 ± 4.07 °C). However, extreme low temperatures (−14.10 °C) were recorded during January and February. Similarly, the highest temperatures occurred in July (25.87 ± 3.26 °C; single highest value was 30.80 °C). Regarding precipitation, the highest monthly values were observed in December (98.65 ± 28.15 mm) and the highest single value was 140 mm in November. The lowest monthly precipitation was recorded in July (29.20 ± 14.40 mm) and the single lowest value (3 mm) was recorded in June or July.

**Figure 3:**
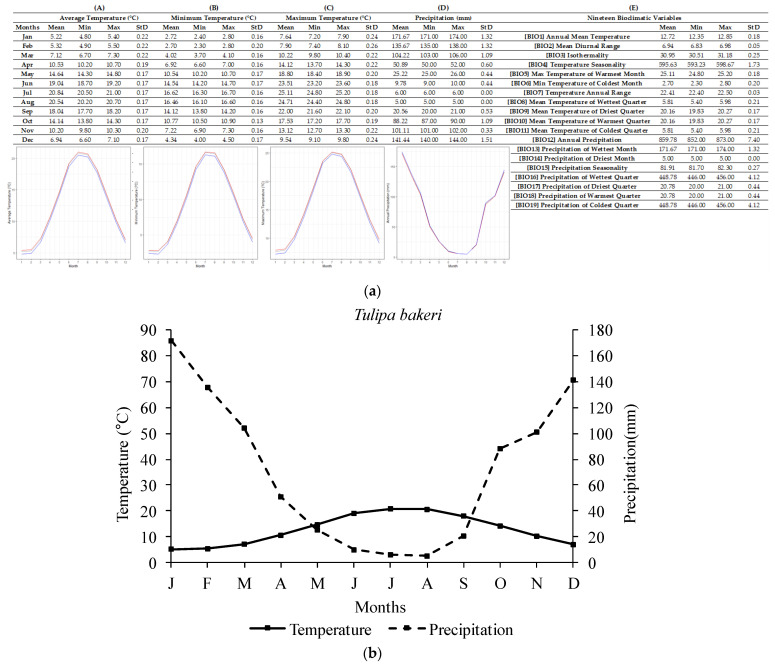
R-derived ecological profile (**a**) and Walter–Lieth climate diagram (**b**) of *Tulipa bakeri* based on nine localities known to date (N = 9). For (**A**–**E**), minimum, maximum, and average values and standard deviations are represented in different columns. Line graphs (**A**–**C**) illustrate the minimum (blue), the maximum (red), and the mean (grey) monthly temperature (°C) and the line graph (**D**) illustrates the minimum (red), maximum (blue), and mean (grey) monthly precipitation (mm). (**A**) shows the minimum temperature in each month (°C), (**B**) shows the maximum temperature in each month (°C), (**C**) shows the average temperatures per month (°C), (**D**) shows the precipitation in each month (mm), and (**E**) shows the values for each of the 19 bioclimatic variables.

The ecological profile of *T. bakeri* (Figure 3) was compiled based on nine available localities of high accuracy, with all localities in Chania, Crete (Greece). For the *T. bakeri* sites, the annual average temperature recorded was 12.72 ± 0.18 °C, with the lowest annual temperature being 12.35 °C and highest being 12.85 °C. The annual average precipitation recorded was 859.78 ± 7.40 mm and the lowest annual value was 852 mm, while the highest one was 873 mm. The lowest temperatures occurred in February (2.70 ± 0.20 °C). The highest temperatures were recorded in July (25.11 ± 0.18 °C). Regarding precipitation, the highest monthly values were recorded in January (171.67 ± 1.32 mm) and the highest single value was 174 mm. The lowest monthly average precipitation was recorded in August (5 ± 0.00 mm) and the single lowest value was 5 mm.

**Figure 4:**
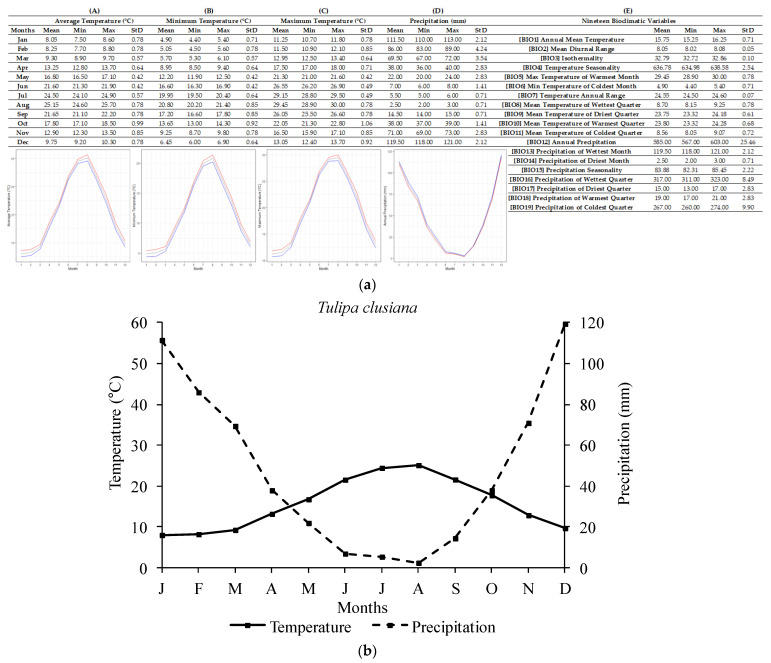
R-derived ecological profile (**a**) and Walter–Lieth climate diagram (**b**) of *Tulipa clusiana* based on two Greek localities known to date (*n* = 2). For (**A**–**E**), minimum, maximum, and average values and standard deviations are represented in different columns. Line graphs (**A**–**C**) illustrate the minimum (blue), the maximum (red), and the mean (grey) monthly temperature (°C) and the line graph (**D**) illustrates the minimum (red), maximum (blue), and mean (grey) monthly precipitation (mm). (**A**) shows the minimum temperature in each month (°C), (**B**) shows the maximum temperature in each month (°C), (**C**) shows the average temperatures per month (°C), (**D**) shows the precipitation in each month (mm), and (**E**) shows the values for each of the 19 bioclimatic variables.

The ecological profile of *T. clusiana* (Figure 4) was compiled based on the only two available Greek localities from Chios Island. For the *T. clusiana* sites, the annual average temperature recorded was 15.75 ± 0.71 °C, with the lowest and highest average annual temperatures being 15.25 °C and 16.25 °C, respectively. The annual precipitation was 585 ± 25.46 mm and the lowest annual value was 567 mm while the highest was 603 mm. The lowest temperatures occurred in January (4.90 ± 0.71 °C; lowest single value of 4.40 °C in January). Similarly, the highest temperatures occurred in August (29.45 ± 0.78 °C; highest: 30 °C). Regarding precipitation, the highest monthly average values were recorded in December (119.50 ± 2.12 mm) and the highest single value was 121 mm. The lowest monthly average precipitation was recorded in August (2.50 ± 0.71 mm; lowest: 2 mm).

**Figure 5:**
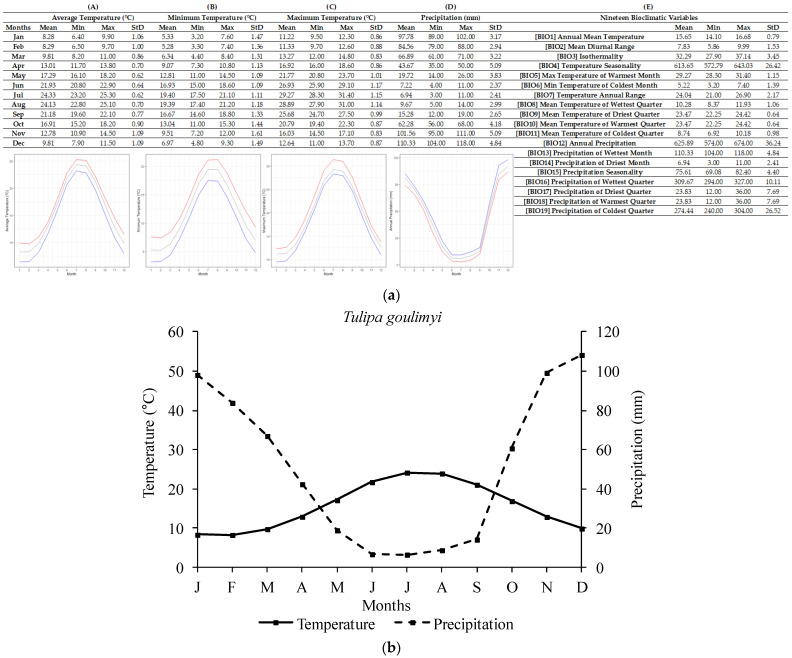
R-derived ecological profile (**a**) and Walter–Lieth climate diagram (**b**) of *Tulipa goulimyi* based on 15 Greek localities of high accuracy (*n* = 15). For (**A**–**E**), minimum, maximum, and average values and standard deviations are represented in different columns. Line graphs (**A**–**C**) illustrate the minimum (blue), the maximum (red), and the mean (grey) monthly temperature (°C) and the line graph (**D**) illustrates the minimum (red), maximum (blue), and mean (grey) monthly precipitation (mm). (**A**) shows the minimum temperature in each month (°C), (**B**) shows the maximum temperature in each month (°C), (**C**) shows the average temperatures per month (°C), (**D**) shows the precipitation in each month (mm), and (**E**) shows the values for each of the 19 bioclimatic variables.

The ecological profile of *T. goulimyi* (Figure 5) was compiled based on 15 localities of high accuracy from South Peloponnese, Kythira Island, and Crete. For the *T. goulimyi* sites, the annual average temperature recorded was 15.73 ± 1.01 °C, with the lowest annual temperature being 14.10 °C and the highest being 17.72 °C. The annual average precipitation recorded was 611.80 ± 34.27 mm, with the lowest and highest annual values being 568 mm and 666 mm, respectively. The lowest temperatures occurred in February (8.47 ± 1.31 °C; lowest single value was 6.50 °C). Similarly, the highest temperatures were recorded in August (24.03 ± 0.72 °C; single highest value was 25.10 °C). Regarding precipitation, the highest monthly values were recorded in December (107.40 ± 4.94 mm) and the highest single value was 113 mm. The lowest monthly average precipitation was recorded in July (6.13 ± 2.85 mm) and the single lowest value (2 mm) was recorded in August.

**Figure 6:**
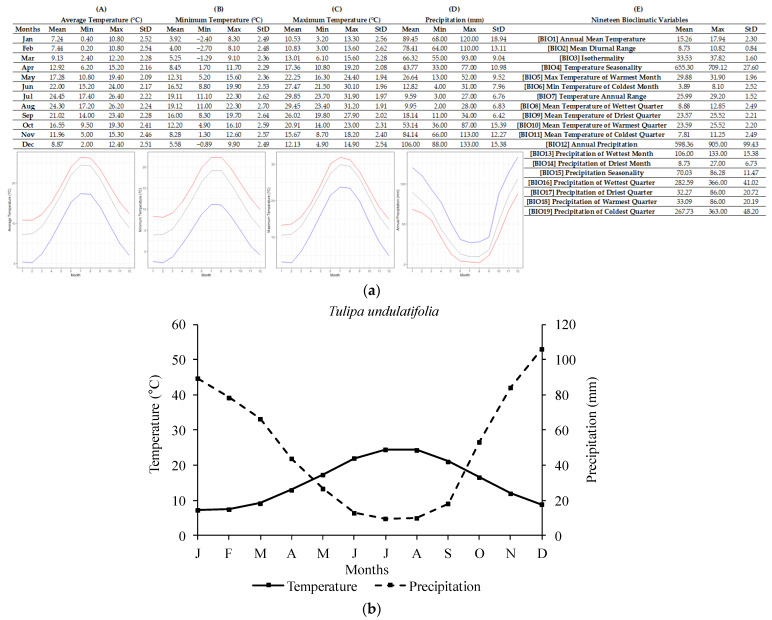
R-derived ecological profile (**a**) and Walter–Lieth climate diagram (**b**) of *Tulipa undulatifolia* based on 22 Greek localities of high accuracy known to date (N = 22). For (**A**–**E**), minimum, maximum, and average values and standard deviations are represented in different columns. Line graphs (**A**–**C**) illustrate the minimum (blue), the maximum (red), and the mean (grey) monthly temperature (°C) and the line graph (**D**) illustrates the minimum (red), maximum (blue), and mean (grey) monthly precipitation (mm). (**A**) shows the minimum temperature in each month (°C), (**B**) shows the maximum temperature in each month (°C), (**C**) shows the average temperatures per month (°C), (**D**) shows the precipitation in each month (mm), and (**E**) shows the values for each of the 19 bioclimatic variables.

The ecological profile of *T. undulatifolia* (Figure 6) was compiled based on 22 localities of high accuracy scattered across mainland and insular Greece. For the *T. undulatifolia* sites, the annual average temperature was 15.30 ± 2.35 °C, with the lowest and highest annual average temperature being 8.35 °C and 17.94 °C, respectively. The annual precipitation recorded was 598.14 ± 101.88 mm (lowest annual value: 506 mm; highest: 905 mm). The lowest temperatures occurred in January (4 ± 2.52 °C) and the lowest single value was recorded in February (−2.70 °C). Similarly, the highest temperatures were recorded in July (29.86 ± 2.01 °C; single highest value was 31.90 °C). Regarding precipitation, the highest monthly average values were recorded in December (106.43 ± 15.62 mm) and the highest single value was 133 mm. The lowest average monthly precipitation was recorded in July (9.14 ± 6.76 mm) and the single lowest value was in August (3 mm).

### 2.2. Seed Germination Tests

**Figure 7:**
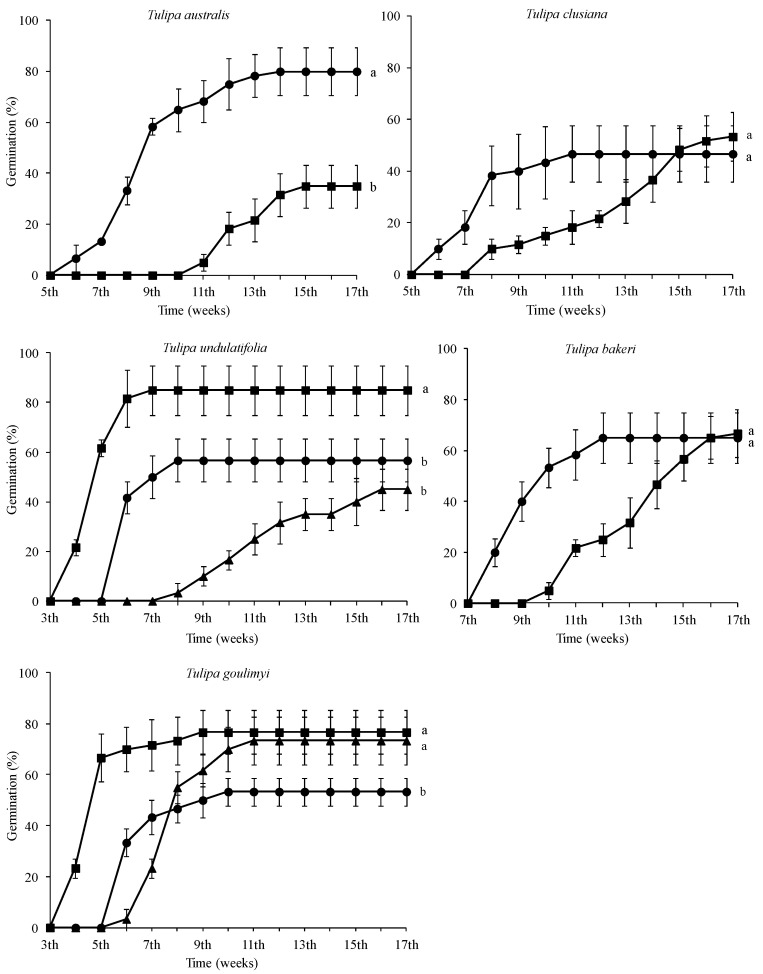
Cumulative seed germination percentage diagrams of Greek *Tulipa australis*, *T. bakeri*, *T. clusiana*, *T. goulimyi*, and *T. undulatifolia* incubated at 5 (●), 10 (■), and 15 °C (▲). In each species, means are statistically different at *p* < 0.05 according to Duncan’s test; values followed by the same letter are not statistically different.

The germination percentage of all tulip species studied herein was affected significantly by temperature (Supplementary Materials, Tables S1–S5). In all five *Tulipa* species, no germination was observed in seeds incubated at 20 and 25 °C. Only *T. undulatifolia* and *T. goulimyi* seeds germinated when incubated at 15 °C (45.00 and 73.33%, respectively) (Figure 7). More precisely, the highest germination in *T. undulatifolia* was observed in seeds incubated at 10 °C (85%), whereas in *T. goulimyi* the highest germination was observed in seeds incubated at 10 and 15 °C (76.67 and 73.33%, respectively). In both species, germination occurred four weeks after the placement of seeds at 10 °C. *Tulipa undulatifolia* seeds completed their germination seven weeks after placement at a temperature of 10 °C, whereas the germination of *T. goulimyi* seeds was completed after nine weeks at a temperature of 10 °C and after 11 weeks when they were kept at 15 °C. The seed germination of *T. bakeri*, *T. australis*, and *T. clusiana* was observed only in seeds incubated at 5 and 10 °C (Figure 7). Seeds of *T. australis* incubated at 5 °C exhibited a higher germination percentage than those incubated at 10 °C. However, no significant differences in germination percentage were observed between *T. bakeri* and *T. clusiana* seeds incubated at 5 and 10 °C. The first germinated seeds of *T. australis* and *T. clusiana* were recorded six weeks after their placement at a temperature of 5 °C, while the germination of seeds incubated at 10 °C was initiated later. The first germinated seeds of *T. bakeri* incubated at 5 and 10 °C were recorded after eight and ten weeks, respectively.

### 2.3. Seedling and Bulblet Production

**Figure 8:**
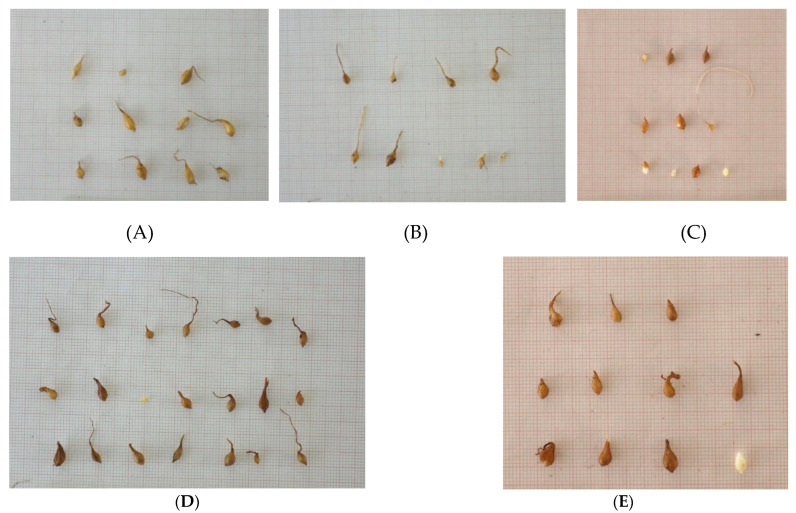
Bulblets produced from seedlings of Greek wild-growing tulips (*Tulipa* spp.) at the end of their first growing season: (**A**) *T. australis*; (**B**) *T. bakeri*; (**C**) *T. clusiana*; (**D**) *T. goulimyi*; (**E**) *T. undulatifolia.*

Significant differences were observed among the bulblet size (weight, length, and width) of the five tulip species (Supplementary Materials, Table S6). The bulblets produced from *T. undulatifolia* and *T. australis* seedlings at the end of their first growing period had a similar weight, which was higher compared to those produced from the other three species. *Tulipa bakeri, T. clusiana*, and *T. goulimyi* had a similar bulblet weight with no significant differences (Table 1). The results for the length of bulblets were also similar (Figure 8, Table 1). No significant differences were observed between the bulblet lengths of *T. undulatifolia* and *T. australis.* These two species produced longer bulblets compared to those produced from the other three species. Regarding bulblet width, the bulblets of *T. undulatifolia* had higher values than those of *T. bakeri*, *T. goulimyi*, and *T. clusiana*. The bulblets of *T. australis* had a larger width compared to those of *T. goulimyi* and *T. clusiana* (Figure 8, Table 1).

## 3. Discussion

This study outlines, for the first time, the ecological requirements of five wild-growing Greek tulip species (*T. australis, T. bakeri, T. clusiana, T. goulimyi*, and *T. undulatifolia*) in terms of temperature and precipitation regimes prevailing in their natural respective ranges in Greece. Therefore, the data generated herein may be perceived as a contribution to the ex situ conservation efforts currently being undertaken for all of the Greek tulip species [3], allowing for, at the same time, the design of appropriate re-introduction actions if deemed necessary (e.g., [25,27]). By contrasting the five ecological profiles of *Tulipa* spp. based on their natural range in Greece (Table 2), *T. bakeri* appeared to have the highest annual temperature preference, and *T. australis* appeared to have the lowest annual temperature requirements. It can be further noticed that *T. australis* and *T. clusiana* are associated with lowest temperatures in January in contrast to *T. bakeri* and *T. undulatifolia*, which are naturally adapted to experience their lowest temperatures in February. *T. undulatifolia* and *T. australis* were the only species able to naturally withstand temperatures below 0 °C (Table 2). Additionally, *T. clusiana* was the only species that had adapted to experience the highest temperatures in August, while the rest of the four species showed the highest temperatures in July. *T. bakeri* was the species with the lowest values for high temperatures and the only one that did not surpass the limit of 30 °C in summer. *T. australis* seemed to be the least resistant species to summer drought and *T. bakeri* seemed to be the species with the highest precipitation demands in the wettest season (Table 2).

This study presents, for the first time, original data regarding the germination of five wild-growing Greek tulip species (*T. australis, T. bakeri, T. clusiana, T. goulimyi*, and *T. undulatifolia*). Therefore, the data generated herein may be perceived as a contribution to the ex situ conservation efforts undertaken for at least three threatened Greek endemic (*T. bakeri* and *T. goulimyi*) or subendemic species (*T. undulatifolia*), allowing for, at the same time, possible re-introduction actions in the future if needed (e.g., [3,25]).

Generally, similar germination patterns were observed in the seeds of all five Greek tulip species studied herein. The results of the present study clearly showed that temperature affected the seed germination of the five studied species. Specifically, the seeds of all five species germinated only at low temperatures (5–15 °C) since a temperature increase to 20–25 °C resulted in the absence of seed germination; this confirmed the initial research hypothesis for the Greek species studied herein, aligning with the previous literature data [2,11]. The lack of germination at high temperatures (20–30 °C) has already been reported for seeds of *T. kaufmanniana* [12]. The germination of seeds of the five species at low temperatures is consistent with previous observations regarding *T. systola* and *T. iliensis* [9,10], which have highlighted that seeds may exhibit the highest germination at a constant temperature of 4 °C, with no seed germination at temperatures higher that 16 °C [2,11]. Furthermore, it is well documented that the seed germination of species from the Mediterranean region is generally observed at relatively low temperatures [18,29,30], suggesting that this behavior should be considered as an adaption of plants to the Mediterranean climate.

In addition, a delay in radicle emergence was observed for all five species, and this delay ranged from four to eight weeks, depending on the studied tulip species. The response to temperature as well as the observed delay in the onset of germination may confirm the existence of some type of dormancy in the seeds of the five Greek tulips studied herein, thus confirming the initial research hypothesis and aligning with previously published data on other *Tulipa* spp. from other regions [2,11,31]. Most likely, the exposure of seeds to low temperatures resulted in the breaking of dormancy and their germination, seedling development, and bulblet production till the end of their biological cycle. Previous studies [31] have reported that seeds of *Tulipa urumiensis* Stapf may present a deep physiological dormancy in addition to morphological dormancy. Furthermore, several Liliaceae members (the family which the members of the genus *Tulipa* belong to) are known to have seeds with an underdeveloped embryo, and therefore, they are associated with either morphological or morphophysiological dormancy [14]. However, more research is needed to determine whether the seeds of these five Greek tulip species have morphological or morphophysiological dormancy. Although it is not currently possible to convey the exact type of morphophysiological dormancy, the observed different responses in temperature among the five species could be attributed to their different degree of dormancy. More precisely, in *T. australis*, *T. bakeri*, and *T. clusiana*, the temperature of 5 °C was more effective than 10 °C, resulting in a better and faster seed germination rate at 5 °C (see Figure 7). The temperature of 10 °C is usually too high to be effective for the cold stratification of seeds [14]. Furthermore, a long time (6–8 weeks) was required for germination onset, and this is probably associated with the expected soil moisture over a long period to ensure successful seed germination in wild habitats. The R-derived ecological profiles can provide significant information regarding the climate conditions under which a plant species grows in its natural habitats [21,22]. According to the ecological profiles of these three tulip species (see Figure 2, Figure 3 and Figure 4), it was shown that such low temperatures (<10 °C) naturally prevail during the winter period in their natural habitats, with additional high soil moisture due to increased precipitation also recorded during this period. These findings suggest that the optimum conditions seem to be met for dormancy breaking and seed germination. Adaptations to local ecological conditions have probably led to differences among the studied species in terms of seed germination requirements. For example, only *T. australis* seeds exhibited the highest germination percentage at 5 °C; this may be a possible adaption which was developed due to the comparatively lower winter temperatures prevailing in the high-altitude natural habitats of *T. australis* compared to those of the other species (see Table 2).

On the other hand, the seeds of *T. undulatifolia* and *T. goulimyi* germinated better at 10 °C, even though germination occurred also at 15 °C. Furthermore, the seeds of both species germinated faster than those of the other three species (see Figure 7). In general, it is known that germination behavior may vary greatly among the species of a given family [14]. According to the ecological profiles generated herein for the different Greek tulips, the seeds of these two species are exposed to optimum conditions for dormancy breaking from late autumn to early winter, where the mean air temperatures in their wild habitats reach about 10 °C and the soil is moist enough due to increased seasonal precipitation (see Figure 5 and Figure 6). In the present study, the first germinated seeds of both *T. undulatifolia* and *T. goulimyi*, which were incubated at 10 °Cc were recorded on the 4th week, thus suggesting that in the natural habitat germination seems to take place until the middle of winter when the seedlings emerge. The seeds of the studied tulip species possibly continue to mature and are probably naturally dispersed during summer. The five tulip species studied herein seem to have developed a seed dormancy mechanism which prevents germination during summer or autumn and allows for germination to take place during the winter. This strategy may ensure that plants will have a longer growing period available until the onset of summer drought.

As far as the bulblets of the five tulip species are concerned, this is the first time that measurements related to their size (weight, length, and width) at the end of the first growing period of seedlings have been presented. Such data may be perceived as a contribution to the ex situ conservation efforts undertaken for at least three threatened species (*T. bakeri, T. goulimyi,* and *T. undulatifolia*), aiming to determine the appropriate period for possible re-introduction actions in the future, but also to facilitate the monitoring of their cultivation in artificial settings (e.g., [3,25]). In the absence of similar data, other studies [32] have shown that the size and diameter of the bulbs after recultivation are usually much larger (from 11–12 cm); however, such data are not comparable with the present study because they refer to asexual propagation. In the present study, small bulblets of these five tulip species were developed from seedlings during their first growing season; they will probably need another three to four years of growth before they reach the critical bulb size to allow flowering [33]. Therefore, long-term ex situ monitoring of the produced bulblets will continue at the premises of the Institute of Plant Breeding and Genetic Resources, Agricultural Organization-Demeter, Thermi, Greece.

## 4. Materials and Methods

### 4.1. Focal Greek Tulips and Seed Collection Data

All *Tulipa* spp. of Greece are protected by the Greek Presidential Decree 67/1981 [3]. The plant nomenclature of the Greek tulip species studied herein follows that used for the Flora of Greece web, version IV (https://portal.cybertaxonomy.org/flora-greece/intro, accessed on 22 February 2023). Three of the Greek tulips are threatened local Greek endemics or Balkan subendemics:

(i) *T. bakeri* is a critically endangered (CR) Greek endemic species [28] found exclusively in Crete (Omalos Plateau, Chania) at altitudes between 700 and 1300 m where it grows in natural and agricultural habitats such as mountain plateaus and field margins, as well as in scrub or by sandy and gravelly stream sides, with a flowering season extending from April to May [34].

(ii) *T. goulimyi* is a vulnerable (VU) Greek endemic species [28] found in Peloponnese, Elafonisos, Kythira, Antikythira, and Crete as a lowland species at altitudes from 0 to 900 m, occurring in natural habitats of terra rossa with phrygana and with flowering during March, April, and May [34].

(iii) *T. undulatifolia* is a vulnerable (VU) wild-growing native species of Greece found in the South Balkan countries and in western and central Turkey (Anatolia) [3]. In Greece, it is mainly found in Peloponnese, Sterea Hellas, and some of the East Aegean Islands (Chios and Lesvos) at altitudes between 100 and 800 m where it grows in soil pockets on rocky and stony slopes with phrygana, in open woodland, or as a weed in cultivated and fallow fields and olive groves, and flowers in March and April [34].

Another two tulips studied herein have a wider distribution range. *T. australis* (is a wild-growing Mediterranean native species found in South European and North African countries eastwards to western Turkey, and in Greece it occurs in the mainland region and some Aegean Islands such as Thasos and Naxos [34] at altitudes of 500–2000 m. It grows in rocky flats, screes, meadows, open woodland, verges of mountain roads, and rocky slopes and flowers in May and June [34]. *T. clusiana* (Figure 1) originates from Asia (Iran to Pakistan) and extends to Greece as a naturalized alien species; it is found in a few lowland (100–600 m) locations of Chios Island, where it grows in agricultural habitats such as seasonally mesic spots of cultivated and fallow fields and flowers in March and April [34].

Fruits (capsules) with mature seeds from about 10 wild-growing individuals of each studied species were collected before being dispersed by hand during botanical expeditions in 2020 and 2021 in the frame of the research project TULIPS.GR (Table 3). The capsules and seeds were collected with special permission (182336/879 on 16 May 2019 64886/2959 on 6 July 2020, and 26895/1527 on 21 April 2021) issued yearly by the national competent authority, namely, the Greek Ministry of Environment and Energy. All collected materials were taxonomically identified and an IPEN (International Plant Exchange Network) code was assigned to them (Table 3). After collection, the seeds of each taxon were manually cleaned, and then they were stored dry in glass containers at 3–5 °C before using them in germination experiments.

**Figure 9:**
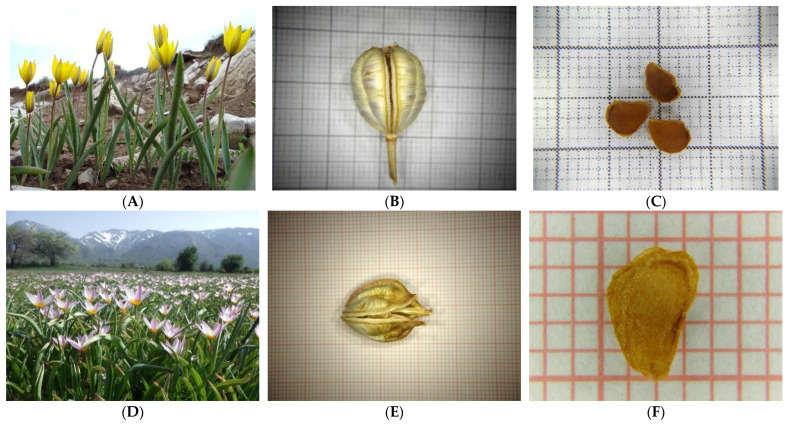

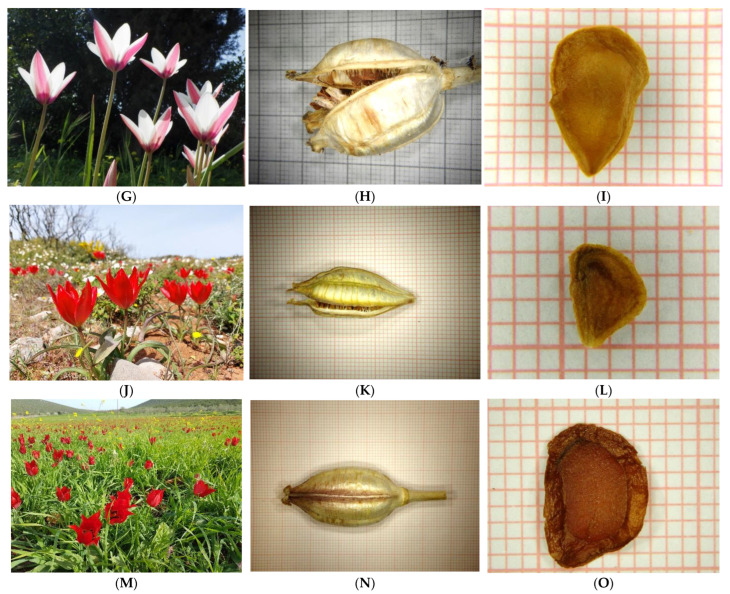
Flowering individuals, mature capsules, and seeds of wild-growing *Tulipa australis* (**A**, **B**, and **C**, respectively), *Tulipa bakeri* (**D**, **E**, and **F**, respectively), *Tulipa clusiana* (**G**, **H**, and **I**, respectively), *Tulipa goulimyi* (**J**, **K**, and **L**, respectively), and *Tulipa undulatifolia* (**M**, **N**, and **O**, respectively) collected from Greece.

Flowering individuals, mature capsules, and seeds of alll wild-growing Greek tulips species studied herein are illustrated in Figure 9.

### 4.2. Distribution Data

In this study, we collected all spatial information referring to the Greek localities of the studied *Tulipa* spp. from the following sources: Lund Virtual Herbarium (http://herbarium.emg.umu.se/, accessed on 6 March 2023), GBIF (https://www.gbif.org/, accessed on 6 March 2023), and JAQC herbaria (https://www.jacq.org/, accessed on 6 March 2023). The data from these online sources were supplemented with unpublished information from the database of the Balkan Botanic Garden of Kroussia and the personal herbaria of Dr. P. Bareka (Department of Crop Science, Agricultural University of Athens; personal communication) and Dr. T. Constantinidis (Department of Biology, National and Kapodistrian University of Athens; personal communication).

Table S7 in the Supplementary Materials includes an overview of the information known to date regarding all Greek localities *of T. australis* (71 localities)*, T. bakeri* (11 localities), *T. clusiana* (2 localities)*, T. goulimyi* (24 localities), and *T. undulatifolia* (24 localities), which were used for mapping their distribution in Greece. The original data were organized and converted where necessary in the WGS84 coordinate system. The mapping of all distribution records for each species was performed in qGIS, illustrating the most updated distribution map for these five *Tulipa* spp. in Greece (Figure 1).

### 4.3. Ecological Data Mining

Based on the Greek distribution localities for each species, we excluded from further use the locations with low spatial accuracy. Therefore, we continued our analysis only with 55 localities of *T. australis* (77.5% of extant Greek localities), 9 localities of *T. bakeri* (81.8%), 2 localities of *T. clusiana* (100%), 14 localities of *T. goulimyi* (58.3%), and 21 localities of *T. undulatifolia* (87.5%). Using these records, we compiled a respective species-specific ecological profile using R’s raster package [35] and WorldClim’s (version 2.1) climate information [36]. The dataset included the linkage of the species’ distribution points with the 19 bioclimatic variables and monthly data about the temperature and precipitation of each locality per species, which was carried out following an approach that has been previously used to determine the ecological preferences of other species [21,22,23,24,27,37]. Following our own previous studies [25,26], we created a stack of raster files consisting of environmental data and extracted numeric information corresponding with the spatial data that we compiled for the distribution of each species (known presence points of the studied *Tulipa* spp.). Finally, we compiled these extracted data in five different factsheets to summarize the ecological preferences of each of the Geek tulip species studied herein by noting the average, minimum, and maximum values prevailing for the original wild habitats of the known populations for each species.

### 4.4. Germination Tests

The germination experiments were initiated in January 2022 and were conducted in the Laboratory of Floriculture, School of Agriculture, Aristotle University of Thessaloniki.

**Figure 10:**
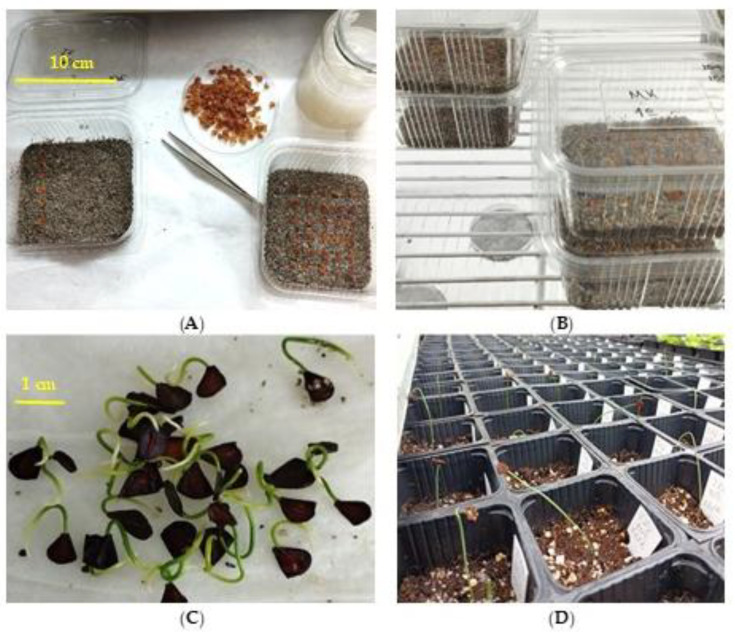
(**A**) Sowing of wild-growing Greek tulip seeds in plastic boxes with disinfected sand consequently placed in a growth chamber (**B**); (**C**) germinated seeds of the vulnerable Balkan subendemic *Tulipa undulatifolia*; (**D**) seedlings of the vulnerable Greek endemic *Tulipa goulimyi* growing in plastic pots in a greenhouse 30 days after germination.

Seed germination of each Greek tulip species was investigated using temperature-controlled growth chambers (CRW-500SD Chrisagis, Athens, Greece). Specifically, their germination responses at five constant temperatures of 5, 10, 15, 20, and 25 °C were evaluated. For each temperature, 4 replications of 15 seeds were used. The seeds were placed in 9 × 10 cm transparent plastic containers on filter paper. To maintain moisture, a layer of moist, sterile sand was placed under the filter paper (Figure 10A). Subsequently, the plastic containers were randomly arranged on the shelves of the growth chambers (Figure 10B), with a 12 h light/dark photoperiod, and the substrate (filter paper and sand) was kept moistened as required during the whole experimental period. The germinated seeds were counted and removed every week for a period of four months. A seed was considered as germinated when at least a 2 mm long radicle had emerged through the seed coat [38].

### 4.5. Seedling Growth

For each wild-growing Greek *Tulipa* species, the germinated seeds were sown in plastic pots (6 × 6 × 6.5 cm) filled with a 3:1 ratio (*v/v*) mixture of enriched peat (TS2, Klassmann) and perlite. The sown seeds were carefully covered with sand and the pots were placed on a bench in the greenhouse without heating. Watering was performed every three days to maintain the proper moisture conditions for seedling growth.

In the seedlings of all five Greek tulip species, integrated nutrient management (INM) was applied. The INM fertilization consisted of a nutrient solution of PRECISE at 0.6 mL/L, THEOCAL at 0.75 g/L, THEOCOPPER at 3.0 mL/L, and Hyper Phos 570 at 0.3 g/L. The fertilizations were started in late of March and were applied every two weeks until the end of May. In total, five fertilizations were applied, and in each application, a constant amount of 3 mL of the above nutrient solution was added to each pot (Figure 10D). Usually, the fertilization was applied a day after seedling irrigation.

In July 2022, the seedlings (cotyledonary leaf) of all five Greek tulip species were desiccated and their one-year-old bulblets were removed from the pots (Figure 8). Subsequently, measurements of the length and width of each bulblet were performed by using a ruler, while the weight was measured using a precision laboratory scale with four decimals.

### 4.6. Statistical Analysis

For the seed germination experiment of each species, a completely randomized design was employed. The comparisons of the means were made using Duncan’s test at a significance level of *p* ≤ 0.05 [39]. Prior to the ANOVA, only the germination percentage data were transformed to arc-sine square root values [40]. All statistical analyses were carried out using SPSS 21.0 (SPSS, Inc., Chicago, IL, USA).

## 5. Conclusions

For the first time, this study demonstrates R-derived detailed ecological profiles of *T. australis*, *T. bakeri*, *T. clusiana*, *T. goulimyi,* and *T. undulatifolia* to provide insight into the abiotic environmental conditions prevailing in the wild habitats of these five wild-growing Greek tulips, thus informing us about the conditions that can be imitated during their ex situ cultivation and acclimatization in man-made environments. Furthermore, the effective sexual propagation of the studied tulip species first reported herein can be perceived as a basic step enabling both in situ conservation and ex situ conservation efforts for species already threatened with extinction (*T. bakeri, T. goulimyi,* and *T. undulatifolia*), or enabling sustainable exploitation strategies for all these popular ornamental plants. Furthermore, the germinated tulip seedlings provide valuable genetic material for current and future micropropagation experiments. The results of the present study indicate a range-specific temperature dependence in the seed germination of the investigated wild-growing Greek tulips, while the differences among species in terms of seed germination requirements could be attributed their adaptation to local ecological conditions.

## Figures and Tables

**Table 1:** Weight, length, and width of *Tulipa australis*, *T. bakeri*, *T. clusiana*, *T. goulimyi*, and *T. undulatifolia* bulblets at the end of the first growing period. Mean ± standard deviation values are provided.

Species	Bulblet Weight (mg)	Bulblet Length (cm)	Bulblet Width (cm)
*Tulipa australis*	25.26 ± 3.76 a	0.2455 ± 0.021 a	0.4909 ± 0.045 ab
*Tulipa bakeri*	11.82 ± 2.50 b	0.1667 ± 0.022 b	0.4333 ± 0.028 bc
*Tulipa clusiana*	15.19 ± 2.31 b	0.1909 ± 0.021 b	0.3545 ± 0.031 d
*Tulipa goulimyi*	14.04 ± 1.72 b	0.1947 ± 0.009 b	0.3842 ± 0.016 cd
*Tulipa undulatifolia*	23.76 ± 2.70 a	0.2583 ± 0.019 a	0.5083 ± 0.023 a

Values in the same column followed by the same letter are not significantly different (*p* > 0.05) according to Duncan’s test.

**Table 2:** Extreme precipitation and temperature limits based on the respective ecological profiles of the five Greek *Tulipa* spp. studied herein (Figure 2, Figure 3, Figure 4, Figure 5 and Figure 6).

**Tulipa spp.**	**Annual Temperature (°C)**	**Lowest Annual Temperature (°C)**	**Highest Annual Temperature (°C)**
*T. australis*	10.43 ± 3.37	4.22	17.80
*T. bakeri*	12.72 ± 0.18	13.35	12.85
*T. clusiana*	15.75 ± 0.71	15.25	16.25
*T. goulimyi*	15.73 ± 1.01	14.10	17.72
*T. undulatifolia*	15.26 ± 2.30	8.35	17.94
**Tulipa spp.**	**Annual Precipitation (mm)**	**Lowest Annual Precipitation (mm)**	**Highest Annual Precipitation (mm)**
*T. australis*	740.15 ± 163.08	455	1026
*T. bakeri*	859.78 ± 7.40	852	873
*T. clusiana*	585 ± 25.46	567	603
*T. goulimyi*	611.80 ± 34.27	568	666
*T. undulatifolia*	598 ± 99.43	506	905

**Table 3:** Seed collection details and IPEN (International Plant Exchange Network) accession numbers regarding the Greek *Tulipa* spp. studied herein.

Scientific Name/Family	IPEN Accession	Altitude (m)	Collection Site and Area	Latitude (North)	Longitude (East)
*Tulipa australis*	GR-BBGK-1- 21,278	1614	Mt. Chelmos, Peloponnese	38.01447	22.19009
*Tulipa bakeri*	GR-BBGK-1- 20,414	1200	Omalos, Chania	35.33333	23.9000
*Tulipa clusiana*	GR-BBGK-1- 21,277	77	Agios Ioannis Prodromos, Chios Island	38.33788	26.09824
*Tulipa goulimyi*	GR-BBGK-1- 20,149	612	Molai, Lakonia	36.82899	22.94770
*Tulipa undulatifolia*	GR-BBGK-1- 20,144	152	Didyma, Argolida	37.45261	23.17304

## Data Availability

All data supporting the results of this study are included in the manuscript, and the datasets are available upon request.

## Supplementary Materials

The following supporting information can be downloaded at: https://www.mdpi.com/article/10.3390/plants12071574/s1, Table S1. ANOVA results concerning the effect of temperature on germination of *Tulipa australis* seeds; Table S2. ANOVA results concerning the effect of temperature on germination of *Tulipa bakeri* seeds; Table S3. ANOVA results concerning the effect of temperature on germination of *Tulipa clusiana* seeds; Table S4. ANOVA results concerning the effect of temperature on germination of *Tulipa goulimyi* seeds; Table S5. ANOVA results concerning the effect of temperature on germination of *Tulipa undulatifolia* seeds; Table S6. Analysis of variance (ANOVA) among the five studied Greek tulip species regarding weight, length, and width of bulblets produced from seeds after the first growing period; Table S7. Spatial (geo-referenced) information of herbarium specimens of *Tulipa australis, T. bakeri, T. clusiana, T. goulimyi*, and *T. undulatifolia* from Lund Virtual Herbarium (http://herbarium.emg.umu.se/, accessed 31 March 2023), GBIF (https://www.gbif.org/, accessed 31 March 2023), JAQC herbaria (https://www.jacq.org/, accessed 31 March 2023), the Balkan Botanic Garden of Kroussia’s database, and the personal herbaria of Dr. P. Bareka (Department of Crop Science, Agricultural University of Athens) and Dr. T. Constantinidis (Department of Biology, National and Kapodistrian University of Athens) used in this study.
