# Supplementary material for: Influence of Temperature on Seed Germination of Five Wild-Growing Tulipa Species of Greece Associated with Their Ecological Profiles: Implications for Conservation and Cultivation
Source: Plants (Basel). 2023 Apr 6;12(7):1574. doi: 10.3390/plants12071574 (PMC10096705; doi:10.3390/plants12071574)
Supplement: Supplementary file 1 [file plants-12-01574-s001.zip › plants-2295215-Supplementary material Table S1.pdf]

Supplementary Material Table S1. ANOVA results concerning the effect of temperature on germination of *Tulipa australis* seeds.

Source	Sum of Squares	df	Mean Square	F	Sig.
Temperatures	13531.67	4	3382.92	249.65	0.000
Error	203.26	15	13.55		
