# Supplementary material for: Influence of Temperature on Seed Germination of Five Wild-Growing Tulipa Species of Greece Associated with Their Ecological Profiles: Implications for Conservation and Cultivation
Source: Plants (Basel). 2023 Apr 6;12(7):1574. doi: 10.3390/plants12071574 (PMC10096705; doi:10.3390/plants12071574)
Supplement: Supplementary file 1 [file plants-12-01574-s001.zip › plants-2295215-Supplementary material Table S2.pdf]

Supplementary Material Table S2. ANOVA results concerning the effect of temperature on germination of *Tulipa bakeri* seeds.

Source	Sum of Squares	df	Mean Square	F	Sig.
Temperatures	14191.89	4	3547.97	259.76	0.000
Error	204.88	15	13.66		
