# Supplementary material for: Influence of Temperature on Seed Germination of Five Wild-Growing Tulipa Species of Greece Associated with Their Ecological Profiles: Implications for Conservation and Cultivation
Source: Plants (Basel). 2023 Apr 6;12(7):1574. doi: 10.3390/plants12071574 (PMC10096705; doi:10.3390/plants12071574)
Supplement: Supplementary file 1 [file plants-12-01574-s001.zip › plants-2295215-Supplementary material Table S3.pdf]

Supplementary Material Table S3. ANOVA results concerning the effect of temperature on germination of *Tulipa clusiana* seeds.

Source	Sum of Squares	df	Mean Square	F	Sig.
Temperatures	9752.66	4	2438.16	173.52	0.000
Error	210.77	15	14.05		
