# Supplementary material for: Influence of Temperature on Seed Germination of Five Wild-Growing Tulipa Species of Greece Associated with Their Ecological Profiles: Implications for Conservation and Cultivation
Source: Plants (Basel). 2023 Apr 6;12(7):1574. doi: 10.3390/plants12071574 (PMC10096705; doi:10.3390/plants12071574)
Supplement: Supplementary file 1 [file plants-12-01574-s001.zip › plants-2295215-Supplementary material Table S5.pdf]

Supplementary Material Table S5. ANOVA results concerning the effect of temperature on germination of *Tulipa undulatifolia* seeds.

Source	Sum of Squares	df	Mean Square	F	Sig.
Temperatures	14952.02	4	3738.01	160.29	0.000
Error	349.79	15	23.32		
