# Supplementary material for: Influence of Temperature on Seed Germination of Five Wild-Growing Tulipa Species of Greece Associated with Their Ecological Profiles: Implications for Conservation and Cultivation
Source: Plants (Basel). 2023 Apr 6;12(7):1574. doi: 10.3390/plants12071574 (PMC10096705; doi:10.3390/plants12071574)
Supplement: Supplementary file 1 [file plants-12-01574-s001.zip › plants-2295215-Supplementary material Table S6.pdf]

Supplementary Material Table S6. Analysis of Variance (ANOVA) among the five studied Greek tulip species regarding weight, length and width of bulblets produced from seeds after the first growing period.

Source	Sum of Squares	df	Mean Square	F	Sig.
Weight	1804.59	4	451.15	5.47	0.001
Error	4951.19	60	82.52		
Length	0.22	4	0.054	5.49	0.001
Error	0.60	60	0.010		
Width	0.07	4	0.018	4.52	0.003
Error	0.24	60	0.004		
